# Long-term impacts of hurricanes on mortality among Medicare beneficiaries: evidence from Hurricane Sandy

**DOI:** 10.3389/fpubh.2025.1523941

**Published:** 2025-08-06

**Authors:** Olivia J. Keenan, Orysya Soroka, David Abramson, Monika Safford, Martin F. Shapiro, Arnab K. Ghosh

**Affiliations:** ^1^Division of General Internal Medicine, Department of Medicine, Weill Cornell Medical College, Cornell University, New York, NY, United States; ^2^School of Global Public Health, New York University, New York, NY, United States

**Keywords:** extreme weather, flooding, climate change, long-term health, mortality risk, older adults

## Abstract

**Objectives:**

Hurricane-related flooding has long-term socioeconomic effects on impacted areas; however, little is known about the long-term health effects on vulnerable, older residents who remain in impacted neighborhoods. We examined mortality rates among older adults who continued living in ZIP Code Tabulation Areas (ZCTAs) impacted by flooding from Hurricane Sandy for up to 5 years after landfall.

**Methods:**

We conducted a propensity-score matched, ZCTA-level ecological analysis post-Hurricane Sandy across the tri-state area (New York City [NYC], New York state excluding NYC [NY], New Jersey [NJ], and Connecticut [CT]). Using multivariable models, we compared all-cause mortality rates between matched flooded versus non-flooded ZCTAs for up to 5 years after Hurricane Sandy’s landfall, among Medicare fee-for-service (FFS) beneficiaries aged 65 years and older who remained in the same ZCTA from 2013 to 2017. Adjusted mortality rate ratios (aMRR) were estimated for each region, controlling for ZCTA-level demographic and socioeconomic factors informed by the socioecological model of disaster recovery.

**Results:**

Before matching, compared to non-flooded ZCTAs, flooded ZCTAs had a higher average Area Deprivation Index (ADI) national rank (20.8 vs. 14.8) and a lower average median household income ($71,587 vs. $89,213). In the matched, adjusted analysis, the Medicare FFS beneficiaries who resided and remained in flood-impacted ZCTAs had a 9% higher risk of all-cause mortality up to 5 years after the event compared to the beneficiaries in ZCTAs not impacted by flooding (aMRR_OVERALL_ 1.09, 95% CI = 1.06–1.12). Adjusted mortality risk varied across geographic regions. In NYC, ZCTAs impacted by flooding had a significant 8% higher risk of long-term mortality up to 5 years after the event (aMRR_NYC_ 1.08, 95% CI = 1.02–1.15). CT also showed a significant 19% higher risk of long-term mortality up to 5 years (aMRR_CT_ 1.19, 95% CI = 1.09–1.31). However, the results for NJ and NY were not significant (aMRR_NJ_: 1.01, 95% CI = 0.97–1.06; aMRR_NY_: 0.96, 95% CI = 0.86–1.07).

**Conclusion:**

ZCTAs impacted by hurricane-related flooding had higher rates of all-cause mortality up to 5 years after the event, but the magnitude of this effect varied by region. These findings highlight the lingering destructive impact of hurricane-related flooding on older adults and underscore the need for long-term, region-specific disaster planning.

## 1 Introduction

Hurricane forecasting models predict increased hurricane intensity and frequency due to rising ocean temperatures and rainfall induced by climate change (1–3). The National Oceanic and Atmospheric Administration (NOAA) categorizes hurricane storm surges as “the greatest threat” during a hurricane event due to severe flooding, which accounts for over half of all direct fatalities from hurricanes in the United States (U.S.) (4). The short-term health impacts of hurricanes and hurricane-related flooding—often within the first weeks or months of landfall—have been well-studied and include morbidity and mortality due to injuries, drowning, infectious disease outbreaks, cardiovascular disease, and mental health disorders (5–13). However, the long-term impacts require further investigation. Studies have revealed the impact of multiple hurricanes, suggesting an increased risk of mortality up to 2 years after hurricane landfall (5, 14). Hurricanes are likely to have long-term impacts on populations (15), including adverse physical health outcomes (e.g., cardiovascular disease, diabetes) (16, 17), mental health effects (18–20), and increased hospitalization rates (21). Adverse long-term health outcomes also disproportionately affect certain vulnerable subgroups, such as older adults, racial minorities, and low-income people (22, 23).

Several studies underscore the vulnerability of older adults in the aftermath of extreme weather events due to clinical, functional, and physiological issues (24–26). Older adults have higher rates of multiple medical comorbidities, functional limitations, and cognitive impairment (27). Therefore, they are more likely to require social support and formal caregiving, including the provision of home health aides. During and after extreme weather events, underlying health conditions may amplify the vulnerabilities of older adults, which may be further exacerbated by limited healthcare access and quality after a hurricane’s landfall, as evidenced by increased emergency department utilization among older adults after Hurricane Sandy (28).

Moreover, post-disaster recovery has been shown to have enduring impacts on the social and built environments over the long term (28–31). Although hurricane-related floods recede quickly, they cause lasting socioecological impacts, including property damage, housing displacement, reconfiguration of public infrastructure, and environmental destruction (15). For example, evidence from Hurricane Katrina highlights that the median time to achieve housing stability after landfall was approximately 3 years (32), while lost economic opportunities were noted after Hurricane Sandy due to the widespread closure of local businesses in flood-impacted areas of New York City (NYC) (33). For older adults, the long-term disruption of social infrastructure due to extreme weather may be particularly damaging because of the importance of aging in place (24). Attachment to place is often amplified among older adults due to vital resources and important lived experiences/memories within the place they reside (34), which may be adversely impacted by climate change-related events (35). Although several reports have described the importance of place-based attachment and enduring social networks as protective factors against post-disaster harm (24, 36, 37), it remains unclear whether these factors provide long-term protection. Moreover, while individually the value of aging in place may be beneficial, individuals and communities that choose to do so have the potential to amplify their collective vulnerability to extreme weather without the necessary infrastructure (e.g., support to ensure resilience) (38).

To capture the complex, ecological nature of post-disaster recovery and its related impacts on health and well-being, Abramson et al. proposed the socioecological model of post-disaster recovery, underscoring the importance of neighborhood-level characteristics in shaping post-disaster recovery outcomes (29). Importantly, this model builds on the sociological perspective of post-disaster recovery, treating recovery as a contested space where the structure of governmental responses (e.g., delivery of post-disaster aid), community displacement, and power dynamics influence the allocation of resources for recovery and rebuilding (39).

To characterize these long-term health impacts, our study tested the hypothesis that there will be higher long-term mortality rates in flood-impacted ZIP Code Tabulation Areas (ZCTAs) compared to non-flood-impacted ZCTAs among older adults who remained in their pre-Sandy residences. Therefore, we examined the long-term mortality of community-based Medicare fee-for-service (FSS) beneficiaries aged 65 years and older whose residential ZCTAs were impacted by flooding caused by Hurricane Sandy. Hurricane Sandy made landfall on the Northeastern United States coast in October 2012. It was a category 3 storm that, in its immediate aftermath, killed 125 people and caused an estimated $87 billion in damages (2024 CPI-Adjusted)—making it one of the costliest storms in U.S. history (40–42). The U.S. states that were most affected included New Jersey (NJ), New York (NY), and Connecticut (CT). Hurricane Sandy was particularly catastrophic because of its coastal flooding and storm surge effects; individuals living in flooded regions in NJ, NY, and CT experienced impacts such as unusable transportation systems, home destruction, drowning risks, and power loss. In addition, most of the geographic areas hit by the storm were densely populated, which resulted in millions of people adversely impacted (30).

Hurricane Sandy presents a unique research opportunity to study the long-term health effects of hurricanes since the affected areas historically have not experienced hurricanes compared to other regions of the United States. It will become increasingly important for healthcare systems to understand the long-term mortality risks associated with hurricane-related flooding, as storms increase in number and severity and begin to impact new geographic regions. This will allow healthcare systems to both proactively prepare before a hurricane and respond effectively afterward to address the long-term healthcare needs of older adults.

## 2 Materials and methods

### 2.1 Study population and design

Informed by the socioecological model of post-disaster recovery (29), we conducted a ZCTA-level ecological retrospective cohort panel study comparing adjusted annual mortality rates in flood-impacted ZCTAs versus non-flood-impacted ZCTAs across New York City (NYC), New York state excluding NYC (NY), New Jersey (NJ), and Connecticut (CT) for up to 5 years after Hurricane Sandy’s landfall.

We used a 20% nationally representative sample of Medicare fee-for-service (FFS) claims data. The study cohort included beneficiaries aged 65 years and older who were continuously enrolled in Medicare Parts A and B beginning in 2012. Beneficiaries were also required to remain in the same ZCTA (data taken from the Medicare Beneficiary Summary File) from 2012 onward (see exclusion cascade in the Supplementary material for additional details). We chose to conduct this analysis at the ZCTA level as it is the smallest administrative unit available for Medicare beneficiaries in the yearly Medicare Beneficiary Summary Files. Therefore, our exclusion criteria were as follows: non-Medicare FFS beneficiaries (i.e., Medicare Advantage beneficiaries), Medicare FFS beneficiaries under the age of 65 (including those eligible for Medicare due to end-stage renal disease and dually eligible Medicare-Medicaid patients under the age of 65), and beneficiaries who did not remain in the same ZCTA for the duration of the study period.

### 2.2 Variables and data sources

#### 2.2.1 Exposure for adjusted analysis

Our exposure variable was the presence or absence of hurricane-related flooding due to storm surges in each ZCTA. We obtained flooding data from the U.S. Geological Survey’s high-water mark maps (1–2 meters) from October 2012. These flood maps represent the actual flooding that occurred during Hurricane Sandy. To determine whether ZCTAs were impacted by flooding, we mapped the flood data onto ZCTA-level shapefiles using a spatial join between the flood map data and ZCTA shapefile boundaries in ArcGIS. To account for spatial differences between exposed and non-exposed ZCTAs, we defined non-exposed ZCTAs as those whose boundaries were within a 10-mile radius of the flooded ZCTA. We justified this choice as approximating neighboring, non-flooded ZIP codes that were adjacent to the boundaries of the floodplain defined by the U. S. Geological Survey. These areas are similar not only in observable characteristics (e.g., population size, urban vs. rural status, and related infrastructure) but also in non-observable characteristics (e.g., social networks) that are defined by the socioecological model of disaster recovery (29).

#### 2.2.2 Dependent variable for adjusted analysis

The dependent variable was the ZCTA-level death count for each quarter-year, with a total of 20 quarters included in the analysis. The date of death was obtained from the yearly Medicare Beneficiary Summary Files. We chose to aggregate death counts within the quarter of each year given the infrequency of death as an outcome.

#### 2.2.3 Covariates

We carefully selected several ecological variables at the ZCTA level that had the potential to confound the effects between hurricane-related flooding and subsequent mortality rates based on Abramson’s socioecological model (29). A summary of these variables is provided in Table 1.

**Table 1:** Demographic and socioeconomic covariates, data source, and years.

Data source	Covariate (ZCTA-level)	Annual or fixed	Years
Medicare fee-for-service claims	Age	Annual	2013–2017
	Average Charlson score	Annual	2013–2017
	Proportion of female beneficiaries	Annual	2013–2017
	Proportion of White beneficiaries	Annual	2013–2017
American Community Survey	Median household income	Annual	2013–2017
	Proportion of individuals aged 65 years and over	Annual	2013–2017
	Proportion of racial minorities*	Annual	2013–2017
	Proportion of rent-occupied units	Annual	2013–2017
	Proportion of individuals residing in the same house as 1 year ago	Annual	2013–2017
	Proportion of overcrowded households	Annual	2013–2017
University of Wisconsin	Area Deprivation Index (ADI)	Fixed	2011–2015

*Removed from the adjusted analysis due to a variance inflation factor (VIF) greater than 4.

We considered characteristics specific to the Medicare FFS cohort in the baseline year and across all years of the study period. These included the mean age of Medicare FFS beneficiaries within each ZCTA, the proportion of female individuals, and the proportion of White beneficiaries. Mindful that pre-hurricane comorbidities are associated with post-hurricane mortality risk, we also included in our analysis the mean Charlson Comorbidity Index (a weighted average of the number of comorbidities), calculated at the ZCTA level both at baseline and for each year.

We also controlled for population-level characteristics found in the American Community Survey 5-year estimates, both at baseline and annually throughout the study period. These annual population-level estimates included median household income, the proportion of residents in overcrowded households, the proportion of residents in rent-occupied units, the proportion of residents aged over 65 years, and the proportion of residents who had lived in the same house 1 year prior.

In addition to controlling for the specific disaster-related socioeconomic status (SES), to account for the aggregate impact of socioeconomic disadvantage prior to the hurricane, we also included each ZCTA’s nationally ranked Area Deprivation Index (ADI) in the model (43). The ADI uses 17 U.S. Census-based factors to assess socioeconomic disadvantage at the area level (43, 44). This measure is static and represents a time period that incorporates the pre-landfall years (2011–2015).

In addition to accounting for these cohort-specific and population-level characteristics at baseline in our analysis, we also separately considered socioeconomic and demographic factors as time-varying confounders. Older adults, racial minorities, and people with low SES generally experience higher mortality rates, which may be exacerbated after emergencies (45, 46). Moreover, flood exposure over time has been shown to exacerbate socioeconomic disadvantage due to loss of economic opportunities and housing instability (33, 47). For example, disparities in post-disaster relief between renters and homeowners are well documented in disaster science literature. These disparities arise due to factors such as limited funding for renters post-storm, renters’ lack of awareness about risks, and additional co-vulnerabilities faced by renters (48, 49). Finally, the likelihood of moving after a disaster may also increase social vulnerability post-disaster, as those with lower incomes in areas with low resilience may be unable to relocate from affected areas (50).

### 2.3 Propensity score matching

Previous studies demonstrate that older adults, racial minorities, and people with low socioeconomic status are also more vulnerable to the long-term health consequences of extreme flooding events due to their increased risk of exposure to flooding (22, 23, 51, 52). For example, compared to other ZCTAs, those with lower pre-flood SES and a higher proportion of non-White residents were more likely to experience Sandy-related flooding (52). To account for these differences, we employed a propensity score matching approach. We estimated the propensity score for the risk of flooding within each region at the ZCTA level using area-level SES covariates at the baseline year via logistic regression. Then, we calculated weights as the inverse of the estimated propensity scores.

### 2.4 Statistical analysis

The baseline characteristics of the cohort at the ZCTA level (both matched and unmatched) were summarized using averages for continuous values and proportions for categorical values, comparing flood-impacted and non-flood-impacted ZCTAs (Tables 2, 3).

**Table 2:** Medicare fee-for-service demographic and associated ZCTA-level characteristics for Sandy-flooded and non-flooded regions, 2013.

	All	Overall		CT		NJ		NY (excluding NYC)		NYC	
		Flooded	Non-Flooded	Flooded	Non-Flooded	Flooded	Non-Flooded	Flooded	Non-Flooded	Flooded	Non-Flooded
# of ZCTAs	959	454	505	71	38	249	159	8	254	126	54
Mean age, years (SD)*	77.0 (1.4)	77.0 (1.4)	77.0 (1.4)	77.4 (1.2)	77.0 (1.4)	76.7 (1.3)	77.0 (1.3)	77.8 (0.9)	76.8 (1.4)	77.3 (1.6)	77.8 (1.3)
Proportion of individuals over 65 years, % (SD)†*	15 (6.9)	14.8 (6.6)	15.2 (7.3)	15.3 (5.0)	14.2 (3.3)	15.6 (7.4)	14.4 (5.7)	16.0 (5.8)	16.3 (7.4)	12.8 (5.2)	12.9 (10.9)
Proportion of female beneficiaries, % (SD)*	58.8 (6.0)	59.1 (5.9)	58.6 (6.0)	57.8 (3.4)	56.6 (6.8)	58.5 (5.1)	59.0 (5.8)	59.2 (5.4)	57.7 (5.8)	60.9 (7.8)	62.8 (5.3)
Proportion of White beneficiaries, %(IQR) **	88.7 (74.6–94.2)	86.7 (69.8–94.5)	89.9 (78.2–93.8)	93.9 (82.6–96.0)	95.1 (93–96.5)	89.4 (79.6–95.5)	88.2 (74.5–91.9)	82.4 (58.8–87.4)	91.8 (85.4–94.7)	71.8 (29.9–85.3)	53.9 (14.4–76.0)
Proportion of racial minorities, ^a^ %(IQR)†**	20.8 (9.5–41.6)	23.5 (10.1–45.8)	18.2 (9.1–36.6)	13.4 (6.9–30.8)	7.7 (3.7–13)	20.5 (7.6–36.8)	21.5 (12.7–40.3)	41.8 (17.3–67.0)	13.7 (7.5–25.4)	43.2 (24.7–68.9)	66.6 (50.2–83.9)
Mean Charlson score (SD)*	1.3 (0.3)	1.4 (0.4)	1.3 (0.3)	1.2 (0.3)	1.2 (0.2)	1.4 (0.3)	1.3 (0.3)	1.3 (0.2)	1.3 (0.2)	1.5 (0.6)	1.5 (0.3)
ADI National Rank ^b^ (IQR)**	16.8 (9.2–27.5)	20.8 (10.7–31.8)	14.8 (8.8–23.0)	24.6 (14.9–39.8)	20.3 (12.7–30.2)	25.6 (16.1–38.2)	18.0 (10.3–32.6)	14.1 (10.2–21.5)	12 (7.7–18.9)	10.7 (4.8–18.9)	14.3 (9.1–17.4)
Proportion of individuals living in overcrowded households, ^c^ % (IQR) †**	1.8 (0.6–4.7)	2.4 (0.7–5.8)	1.4 (0.5–3.8)	1.0 (0.5–2.8)	0.4 (0.0–1.7)	1.5 (0.4–3.5)	1.3 (0.3–3.7)	5.4 (2.4–9)	1.3 (0.5–2.6)	6.5 (3.5–9.8)	8.4 (5.9–13)
Median Household Income USD (SD)†*	80,869 (32,916)	71,587 (30,306)	89,213 (32,963)	81,286 (36,404)	98,035 (38,274)	71,281 (24,541)	88,691 (33,146)	75,183 (27,912)	95,918 (29,415)	66,498 (35,599)	53,006 (17,224)
Proportion of renters, % (IQR) †**	26.5 (14.5–50.6)	34.4 (19.2–63.6)	21.2 (12.5–35.8)	23.4 (15.0–44.6)	13.6 (10.1–23.7)	26.9 (15.9–44.0)	20.7 (13.2–36.7)	39.9 (22.7–61.2)	18.6 (11.1–28.7)	70.3 (51.2–85.0)	63.7 (44.1–77.2)
Proportion of individuals residing in the same house as one year ago, % (IQR) †**	91.3 (88.1–93.8)	92.4 (89.7–94.4)	90.1 (86.4–92.8)	92.4 (89.6–93.9)	89.4 (86.1–92.0)	92.2 (89.7–94.1)	90.7 (87.4–93.3)	93.0 (90.3–95.0)	90.8 (87.6–94.1)	90.4 (88.3–92.0)	88.7 (84.5–91.4)

SD, standard deviation. IQR, interquartile range. *Characteristics presented as mean (SD) across ZCTAs that belong to a specific region. ** Characteristics presented as median (IQR) across ZCTAs that belong to a specific region. † ZCTA characteristics obtained from ACS-2013 (American Community Survey 5-year estimates: years 2009–2013).

a Racial minority: non-White people.

b ADI: Area Deprivation Index in 2015 (calculated using ACS 2011–2015 5-year averages of 17 socioeconomic disadvantage measures, such as income, education, employment, and housing quality). Lower ADI = less vulnerable population. University of Wisconsin School of Medicine and Public Health. Downloaded from https://www.neighborhoodatlas.medicine.wisc.edu/ 4.10.2024. Neighborhood Atlas - Download Data (wisc.edu).

c Overcrowded households are defined as housing units with more than one occupant per room. The denominator is the number of occupied housing units.

**Table 3:** Medicare fee-for-service demographic and associated ZCTA-level characteristics for Sandy-flooded and non-flooded regions after inverse probability weighting, 2013.

	Overall		CT		NJ		NY (excluding NYC)		NYC	
	Flooded	Non-flooded	Flooded	Non-flooded	Flooded	Non-flooded	Flooded	Non-flooded	Flooded	Non-flooded
Mean age, years (SD)*	77 (2)	76.9 (2.5)	77.3 (1.5)	77.4 (2.1)	76.9 (1.7)	77 (2)	77.9 (1.8)	76.9 (1.4)	77.4 (1.8)	77.5 (2.1)
Proportion of individuals over 65 years, % (SD)†*	14.9 (9)	14.8 (10)	14.9 (5.5)	14.9 (3.5)	15.2 (8.8)	15.7 (15.1)	16 (14.9)	16 (5.6)	12.8 (5.9)	12.6 (15.8)
Proportion of female beneficiaries, %(SD)*	58.9 (7.1)	59.1 (7.7)	57.7 (4.5)	57.8 (7.4)	58.5 (6.2)	58.9 (9.2)	58.6 (16.7)	57.6 (5.3)	62.1 (6.8)	62.9 (6.6)
Proportion of White beneficiaries, %(IQR) **	89 (74.6–95)	88.6 (76.3–93.3)	95.1 (89.2–96.7)	94.3 (92–96.4)	88.6 (76–94.5)	88.6 (77.3–92.4)	85.7 (85.7–85.7)	91.7 (84.4–94.7)	68 (27.1–83.5)	65.5 (23.3–77.9)
Mean Charlson score (SD)*	1.3 (0.4)	1.3 (0.4)	1.2 (0.3)	1.2 (0.3)	1.3 (0.4)	1.3 (0.4)	1.3 (0.7)	1.3 (0.2)	1.5 (0.4)	1.5 (0.5)
ADI National Rank ^a^ (IQR)**	17.9 (8–27.9)	16.8 (9.7–26)	24.1 (14.9–30.7)	20.2 (11.8–29.9)	23.7 (13–35.9)	23.9 (14.8–38.5)	12.4 (12.4–12.4)	12.1 (7.9–19)	12.3 (6.3–19.3)	12.7 (6.5–17.1)
Proportion of individuals living in overcrowded households, ^b^ % (IQR) †**	1.7 (0.4–4.7)	1.7 (0.5–4.2)	0.8 (0.4–1.7)	0.4 (0–2.1)	1.5 (0.3–3.5)	1.5 (0.5–3.3)	2.8 (2.4–2.8)	1.3 (0.5–2.9)	6.9 (3.8–10.7)	6.8 (4.2–11.3)
Median Household Income USD (SD)†*	83,392 (55,513)	81,418 (42,409)	88,905 (46,725)	99,258 (51,071)	78,769 (38,387)	79,483 (45,460)	94,467 (79,854)	95,997 (28,981)	62,542 (38,654)	56,060 (30,996)
Proportion of renters, % (IQR) †**	25.6 (13.8–51.2)	28.5 (15.5–53.5)	17 (12.4–31.5)	17.3 (10.9–27.1)	24.8 (12.4–40.8)	26.3 (14.2–37.2)	14.5 (14.5–17.7)	19.1 (11.2–29.7)	68.9 (50.4–86.4)	69.6 (53.6–83.9)
Proportion of individuals residing in the same house as 1 year ago, % (IQR) †**	91.2 (88.1–93.9)	91.3 (87.6–93.6)	90.9 (87.6–92.9)	91.6 (88.1–93.4)	91.4 (88.1–94.1)	91.8 (88.9–93.4)	93.9 (93.9–93.9)	92.9 (90.3–95)	88.9 (85.4–91.5)	89.4 (86.8–91.7)

a ADI: Area Deprivation Index in 2015 (calculated using ACS 2011–2015 5-year averages of 17 socioeconomic disadvantage measures, such as income, education, employment, and housing quality). Lower ADI = less vulnerable population. University of Wisconsin School of Medicine and Public Health. Downloaded from https://www.neighborhoodatlas.medicine.wisc.edu/ 4.10.2024. Neighborhood Atlas - Download Data (wisc.edu).

b Overcrowded households are defined as housing units with more than one occupant per room. The denominator is the number of occupied housing units.

SD, standard deviation. IQR, interquartile range. *Characteristics presented as mean (SD) across ZCTAs that belong to a specific region. ** Characteristics presented as median (IQR) across ZCTAs that belong to a specific region. † ZCTA characteristics obtained from ACS-2013 (American Community Survey 5-year estimates: years 2009–2013).

**Figure 1:**
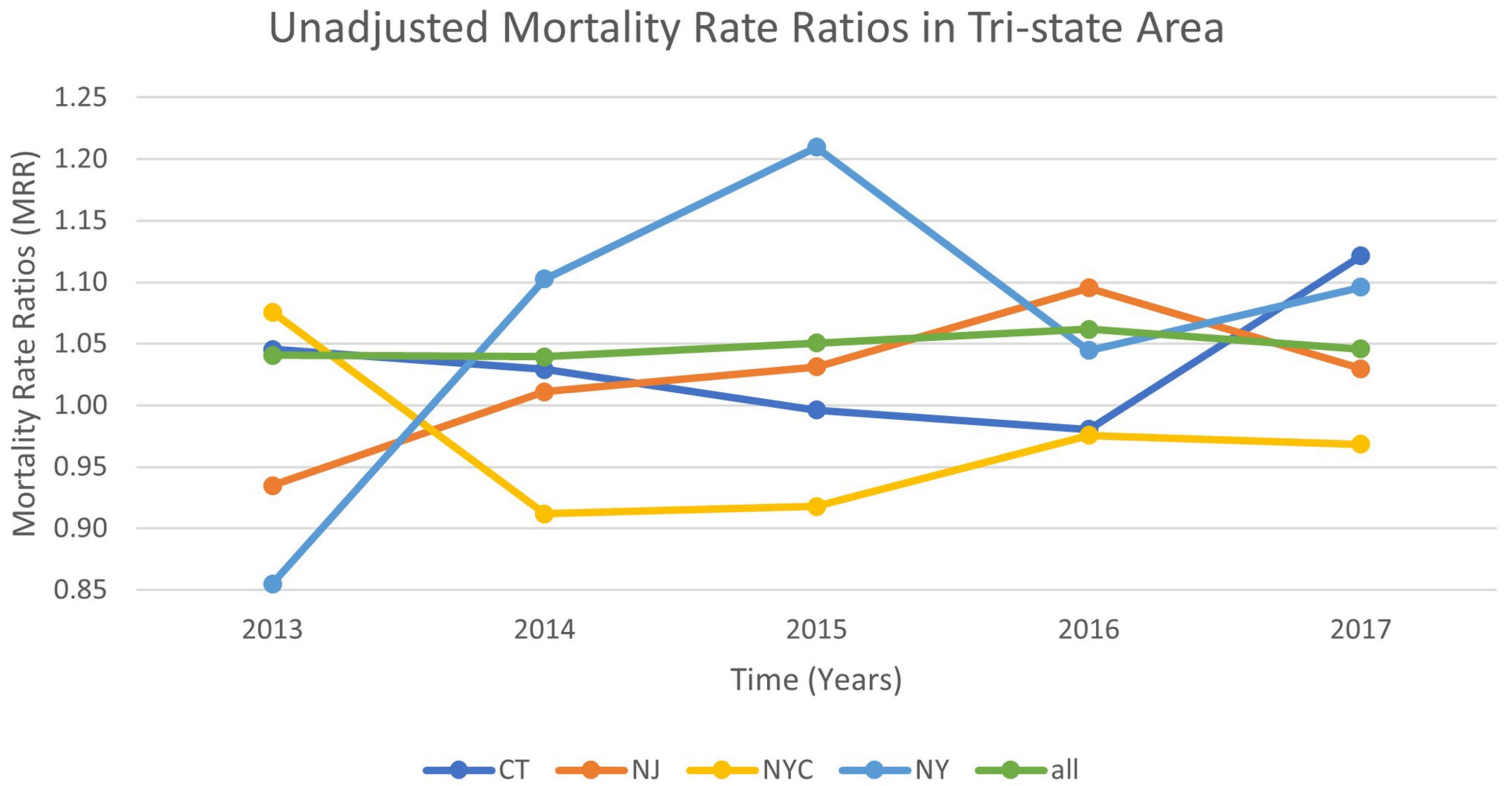
Annual unadjusted mortality rate ratios for the study participants* residing in flood-impacted versus non-impacted ZCTAs after Hurricane Sandy, 2013–2017 *Study participants: Medicare beneficiaries aged 65 years and older in 2012, continuously enrolled in Medicare Parts A and B, who resided in the same ZCTAs for the five-year follow-up period or until death.

To examine differences in unadjusted mortality rates between flood-impacted and non-flood-impacted ZCTAs, we calculated annual unadjusted mortality rates from 2013 to 2017 for all regions combined and separately for NJ, CT, NYC, and NY. We used linear regression to determine the *p*-value of the trend line slope overall and for each region (Figure 1).

Then, we examined ZCTA-level mortality rates after the hurricane’s landfall using aggregated quarter-year measures (2013–2017). Although Hurricane Sandy made landfall in October 2012, we defined the long-term post-exposure period as beginning in the first quarter of 2013. This approach was used to avoid the well-known rise in mortality rates in the 2–3 week period after Sandy’s landfall, putatively due to the acute impacts of the storm (53, 54). Our analysis aimed to investigate a different mechanism: the long-term ecological changes resulting from hurricane-related flooding.

We used a zero-inflated multivariable negative binomial regression to compare the adjusted mortality risk between flooded and non-flooded ZCTAs 5 years after landfall. We controlled for the aforementioned population-level and cohort-specific variables with “stepwise” regression models. We employed two modeling strategies. The first utilized only the baseline covariates from 2013. The second employed these covariates as time-varying where possible. The final model included weights. Multicollinearity was assessed using the variance inflation factor (VIF), and covariates with a VIF greater than 4 were removed. The results of the final model are presented in Table 4 (other model outputs can be found in the Supplementary material). Significance was set at a *p*-value of <0.05; all tests were two-sided.

**Table 4:** Adjusted* mortality rate ratios (aMRR) at 5 years after Hurricane Sandy’s landfall for Medicare FFS beneficiaries who stayed in matched flood-impacted versus non-flood-impacted ZCTAs.

	Adjusted for only baseline covariates		Adjusted for time-varying covariates	
	aMRR (95% CI)	*P*-value	aMRR (95% CI)	*P*-value
All ZCTAs	1.09 (1.06–1.12)	**<0.0001**	1.09 (1.06–1.12)	**<0.0001**
New Jersey (NJ)	1.01 (0.97–1.06)	0.64	0.99 (0.94–1.03)	0.59
Connecticut (CT)	1.19 (1.09–1.31)	**0.0002**	1.18 (1.08–1.30)	**0.0004**
New York (excluding NYC) (NY)	0.96 (0.86–1.07)	0.48	0.97 (0.87–1.08)	0.53
New York City (NYC)	1.08 (1.02–1.15)	**0.009**	1.10 (1.04–1.17)	**0.002**

*Adjusted, at the ZCTA level for annual Medicare fee-for-service cohort characteristics (i.e., proportion of female beneficiaries, proportion of White beneficiaries, mean age, and mean Charlson comorbidity score), annual population-level estimates from the American Community Survey (i.e., proportion of individuals aged 65 and over, proportion of individuals living in overcrowded housing, median household income, proportion of renters, proportion of individuals residing in the same house as 1 year ago), and the combined 2011–2015 ADI national rank. Bold represents *p*-values significant to an alpha of 0.05.

The study protocol was approved by the Weill Cornell Medicine Institutional Review Board on 9 August 2023. All analyses were conducted using SAS version 9.4 (SAS Institute, Inc., Cary, NC, USA).

## 3 Results

### 3.1 Characteristics of the study population at the ZCTA level

Our cohort consisted of 298,271 Medicare FFS beneficiaries aged 65 and older from 959 ZCTAs across NYC, NY, NJ, and CT that were either flooded or located within a 10-mile radius of flooded ZCTAs. Across the study region, 454 ZCTAs were impacted by flooding from Hurricane Sandy in October 2012.

In Table 2, we present the overall and regional ZCTA-level characteristics for flooded and non-flooded ZCTAs in 2013. The average age of the beneficiaries was similar between the flood-impacted and non–flood-impacted ZCTAs (flood-impacted: 77.0 years; non-flood-impacted 77.0 years). Furthermore, the flood-impacted and non-flood-impacted ZCTAs had similar proportions of female individuals (flood-impacted: 59.1%; non-flood-impacted 58.6%) and Charlson morbidity scores (flood-impacted: 1.4; non-flood-impacted 1.3). Greater variation was observed in the ZCTA socioeconomic characteristics, with the non-impacted group having a lower ADI rank (non-flood-impacted: 14.8 vs. flood-impacted: 20.8), lower proportions of overcrowded households (non-flood-impacted: 1.4% vs. flood-impacted: 2.4%), higher median income (non-flood-impacted: $89,213 vs. flood-impacted: $71,587), and lower proportions of renters (non-flood-impacted: 21.2% vs. flood-impacted: 34.4%).

After applying inverse probability weights (Table 3), differences in baseline characteristics were all insignificant, except for differences in the mean age of the beneficiaries in the flooded versus non-flooded ZCTAs in NY (*p*: <0.001). *p*-values for all regions can be found in the Supplementary material.

### 3.2 Unadjusted mortality rates by region over time

Figure 1 demonstrates the unadjusted annual mortality rates for the flooded versus non-flooded ZCTAs over time (from 2013 to 2017) among the Medicare FFS beneficiaries who remained in the same ZCTA after Hurricane Sandy’s landfall. The results from this analysis demonstrated that, across all regions, there was no significant increase or decrease in the mortality trend over the five-year period. *p*-values for the trend across all regions were greater than 0.05 (Overall: 0.32, Connecticut [CT]: 0.63, New Jersey [NJ]: 0.14, New York City [NYC]: 0.55, New York state excluding NYC [NY]: 0.37).

### 3.3 Results from the adjusted analysis

Table 4 shows the results of the fully adjusted models after propensity score matching: the first model included only baseline characteristics, while the second included time-varying characteristics. After controlling for baseline ZCTA-level demographic and socioeconomic factors across all regions, the beneficiaries who resided and stayed in ZCTAs impacted by Hurricane Sandy flooding in the tri-state area (NY, NJ, and CT) had a significant 9% higher risk of long-term mortality up to 5 years compared to the similar beneficiaries in non-flooded ZCTAs (aMRR_OVERALL_ 1.09, 95% CI = 1.06–1.12).

Geographic variation was noted between regions. NYC ZCTAs impacted by flooding had a significant 8% higher risk of long-term mortality up to 5 years (aMRR_NYC_ 1.08, 95% CI = 1.02–1.15). CT also showed a significant 19% higher risk of long-term mortality up to 5 years (aMRR_CT_ 1.19, 95% CI = 1.09–1.31). However, the results for NJ and NY were not significant (aMRR_NJ_: 1.01, 95% CI = 0.97–1.06; aMRR_NY_: 0.96, 95% CI = 0.86–1.07). The results from the model that utilized time-varying characteristics were nearly identical. A full list of model outputs can be found in the Supplementary material.

## 4 Discussion

In this study examining long-term mortality rates among Medicare FFS beneficiaries residing in ZCTAs impacted by flooding from Hurricane Sandy, we found that the overall adjusted mortality risk for older adults was 9% higher at 5 years in flooded regions compared to non-flooded regions. Moreover, regional variation was observed, with flooded ZCTAs in CT and NYC demonstrating the greatest increases in risk—19 and 8% higher, respectively—compared to non-flooded ZCTAs.

The effect size of the significant overall adjusted 9% mortality risk found in this study is in line with previous studies that evaluated post-hurricane mortality risk. Quast et al. (16) found that older individuals with diabetes residing in counties affected by Hurricane Katrina had a significant 8% higher all-cause mortality risk at 5 years compared to those who were unaffected. Another study, Bell et al. (55), found that the mortality risk 1 year after hurricane exposure among Alzheimer’s disease and related dementias (ADRD) populations (<65 years) (defined as having a FEMA disaster declaration) was 10.9% higher in counties exposed to Hurricane Harvey and 6.2% higher in counties exposed to Hurricane Irma compared to counties that were unexposed. Our findings, based on data from continuously enrolled Medicare beneficiaries who remained in place, thus emphasize the specific long-term risks to older adults after hurricanes, especially for those who stay in place, at a finer spatial resolution (37).

The socioecological model of post-disaster recovery underscores the role of socioeconomic disadvantage in limiting post-disaster resilience and long-term health outcomes (29). Low-income individuals in flooded areas experience structural disadvantages to such a degree that they can impact their health and well-being in the long term. For example, Hernández et al. (2018) found that Hurricane Sandy uniquely impacted public housing residents by limiting access to healthcare and transportation, while also exacerbating existing challenges such as energy and housing insecurity; for many residents, these challenges contributed to delayed recovery (56).

In line with previous studies identifying disproportionate flooding exposure post-Hurricane Sandy among low-income populations (52), our study found that hurricane-related flooding in NYC disproportionately impacted low-income neighborhoods, as defined at the ZCTA level. NYC ZCTAs exhibited socioeconomic and demographic characteristics previously associated with greater exposure (23), such as the highest proportion of racial minorities and the lowest household income, compared to the other regions (NJ, CT, and NY). In our analysis, these NYC-flooded ZCTAs had significantly higher mortality risk than non-flooded regions compared to NJ and NY, even after adjusting for differences in exposure risk.

By contrast, CT ZCTAs had a higher proportion of White individuals, higher median household incomes, and the lowest proportion of individuals living in overcrowded households compared to all regions. However, CT had the highest mortality risk in flooded ZCTAs versus non-flooded ZCTAs compared to any other region. ZCTAs in NJ and NY had sociodemographic characteristics in between NYC and CT values and exhibited no difference in mortality risk between non-impacted and impacted ZCTAs. This finding highlights the importance of recognizing the complexity of region-specific outcomes—socioeconomic and demographic characteristics alone may not explain the full post-recovery story. There may be other regional nuances that influence long-term health effects post-flood, such as region-specific policies, infrastructure disruption, and disaster relief—all of which warrant further investigation.

There are limitations to our study. First, we conducted only an ecological analysis. Therefore, we are not able to extend these findings to individual mortality risk because understanding the true complexity of disaster recovery and long-term effects requires analyzing both individual- and ecological-level impacts. Nonetheless, systems of disaster preparedness are often ecological in nature (i.e., focused on populations at risk), and thus, understanding the ecological impact has important ramifications for potential strategies in hurricane-related communication, public health responses pre- and post-disaster, and federal and state responses to disasters. Second, our analysis was limited because the area-level SES data from the ADI were not time-varying. Previous studies have shown time-varying social and economic neighborhood-level impacts of hurricanes, including Hurricane Sandy (30, 33). Third, our sample included a 20% national sample of Medicare FFS beneficiaries. Therefore, we excluded Medicare Advantage populations from our analysis, which constitute up to 54% of Medicare enrollees (57). Finally, we examined beneficiaries who remained in the same ZCTA for the entire post-hurricane exposure period. We did not consider those who left. The implications of this are unclear. While data suggest that individuals who can and have the means to leave post-disaster regions exist, particularly among older adults, the desire to remain in place (related to aging in place and place-based attachment) may offset this risk (37). To assess and compare mortality risks within ZCTAs, our study design required us to only consider individuals who stayed in the post-hurricane setting. Despite this limitation, our finding of higher mortality rates in flood-impacted ZCTAs still emphasizes the vulnerability of older adults who stay in place in impacted areas.

Climate-amplified hurricanes and hurricane-related floods are expected to increase in the future, making it crucial from both health systems and policy perspectives to understand the long-term health effects on those who are most vulnerable. As we write this article, the unprecedented flooding brought on by Hurricane Helene has devastated inland areas of the United States, further demonstrating the changing risk of hurricanes and the urgent need for long-term post-disaster preparation (58). Urban areas such as NYC and the tri-state region (NY, NJ, CT) face unique challenges due to high population density, coastal proximity, and public infrastructure vulnerable to flood damage (public transportation, public housing, and energy) (59). More research is needed to understand which populations are most vulnerable on a regional basis, as well as the socioecological factors at both the individual and neighborhood levels influencing long-term vulnerability. At the individual level, future research should continue to evaluate the mechanisms driving long-term mortality related to hurricanes and hurricane-related flooding. For example, chronic stress related to flooding likely increases the risk of cardiovascular disease (CVD), but little is known about long-term CVD risk post-disaster (13), as well as other clinical maladies affecting long-term mortality post-disaster. At the neighborhood level, future research should incorporate spatiotemporal modeling methods to further elucidate the relationships between long-term mortality risk and flooding across regions over time. Lastly, future post-disaster interventions should prioritize environmental justice and use mixed methods approaches to address the structural and distributive factors influencing long-term health outcomes. These interventions must also prioritize the perspectives of those most affected in their design and address the region-specific impacts highlighted in our analysis.

## Data Availability

The data analyzed in this study is subject to the following licenses/restrictions: data from the Centers of Medicare and Medicaid are not publicly available. Requests to access these datasets should be directed to https://resdac.org.
