# Supplementary material for: Long-term impacts of hurricanes on mortality among Medicare beneficiaries: evidence from Hurricane Sandy
Source: Front Public Health. 2025 Aug 6;13:1523941. doi: 10.3389/fpubh.2025.1523941 (PMC12364919; doi:10.3389/fpubh.2025.1523941)
Supplement: Supplementary file 1 [file Table_1.docx]

Supplementary Material

**Supplementary Table 1.** Table 2B Fee-for-service demographic and associated ZCTA-level characteristics for Sandy-flooded and non-flooded regions with the associated p-values after inverse probability weighting analysis. For several ZCTAs, some covariates were missing so number of ZCTAs is slightly different compared to Table 2A and Table 2B.

|  | **Overall** | |  | **CT** | |  | **NJ** | |  |
| --- | --- | --- | --- | --- | --- | --- | --- | --- | --- |
|  | **Flooded** | **Non-Flooded** | **p-value** | **Flooded** | **Non-Flooded** | **p-value** | **Flooded** | **Non-Flooded** | **p-value** |
| **# ZCTAs** | **453** | **501** |  | **71** | **37** |  | **249** | **159** |  |
| Mean age, years (SD)* | 77 (2) | 76.9 (2.5) | 0.28 | 77.3 (1.5) | 77.4 (2.1) | 0.95 | 76.9 (1.7) | 77 (2) | 0.52 |
| Proportion over 65 years, % (SD)†* | 14.9 (9) | 14.8 (10) | 0.78 | 14.9 (5.5) | 14.9 (3.5) | 0.96 | 15.2 (8.8) | 15.7 (15.1) | 0.53 |
| Proportion female, %(SD)* | 58.9 (7.1) | 59.1 (7.7) | 0.64 | 57.7 (4.5) | 57.8 (7.4) | 0.91 | 58.5 (6.2) | 58.9 (9.2) | 0.48 |
| Proportion White, %(IQR)** | 89 (74.6-95) | 88.6 (76.3-93.3) | 0.78 | 95.1 (89.2-96.7) | 94.3 (92-96.4) | 0.49 | 88.6 (76-94.5) | 88.6 (77.3-92.4) | 0.978 |
| Mean Charlson score (SD)* | 1.3 (0.4) | 1.3 (0.4) | 0.79 | 1.2 (0.3) | 1.2 (0.3) | 0.47 | 1.3 (0.4) | 1.3 (0.4) | 0.86 |
| ADI National Rank ^a^ (IQR)** | 17.9 (8-27.9) | 16.8 (9.7-26) | 0.34 | 24.1 (14.9-30.7) | 20.2 (11.8-29.9) | 0.35 | 23.7 (13-35.9) | 23.9 (14.8-38.5) | 0.92 |
| Proportion lived in overcrowded households, ^b^ %(IQR)†** | 1.7 (0.4-4.7) | 1.7 (0.5-4.2) | 0.99 | 0.8 (0.4-1.7) | 0.4 (0-2.1) | 0.33 | 1.5 (0.3-3.5) | 1.5 (0.5-3.3) | 0.99 |
| Median household income USD (SD)*† | 83,392 (55,513) | 81,418 (42,409) | 0.38 | 88,905 (46,725) | 99,258 (51,071) | 0.14 | 78,769 (38,387) | 79,483 (45,460) | 0.81 |
| Proportion renters, % (IQR)†** | 25.6 (13.8-51.2) | 28.5 (15.5-53.5) | 0.21 | 17 (12.4-31.5) | 17.3 (10.9-27.1) | 0.95 | 24.8 (12.4-40.8) | 26.3 (14.2-37.2) | 0.64 |
| Proportion resided in the same house one year ago, % (IQR) †** | 91.2 (88.1-93.9) | 91.3 (87.6-93.6) | 0.81 | 90.9 (87.6-92.9) | 91.6 (88.1-93.4) | 0.6 | 91.4 (88.1-94.1) | 91.8 (88.9-93.4) | 0.5 |

|  | **NY (excluding NYC)** | |  | **NYC** | |  |
| --- | --- | --- | --- | --- | --- | --- |
|  | **Flooded** | **Non-Flooded** | **p-value** | **Flooded** | **Non-Flooded** | **p-value** |
| **# ZCTAs** | **8** | **252** |  | **125** | **53** |  |
| Mean age, years (SD)* | 77.9 (1.8) | 76.9 (1.4) | <0.001 | 77.4 (1.8) | 77.5 (2.1) | 0.76 |
| Proportion over 65 years, % (SD)†* | 16 (14.9) | 16 (5.6) | 0.95 | 12.8 (5.9) | 12.6 (15.8) | 0.85 |
| Proportion female, % (SD)* | 58.6 (16.7) | 57.6 (5.3) | 0.49 | 62.1 (6.8) | 62.9 (6.6) | 0.25 |
| Proportion White, % (IQR)** | 85.7 (85.7-85.7) | 91.7 (84.4-94.7) | 0.77 | 68 (27.1-83.5) | 65.5 (23.3-77.9) | 0.83 |
| Mean Charlson score (SD)* | 1.3 (0.7) | 1.3 (0.2) | 0.21 | 1.5 (0.4) | 1.5 (0.5) | 0.56 |
| ADI National Rank ^a^ (IQR)** | 12.4 (12.4-12.4) | 12.1 (7.9-19) | 0.89 | 12.3 (6.3-19.3) | 12.7 (6.5-17.1) | 0.84 |
| Proportion lived in overcrowded households ^b^ %(IQR)†** | 2.8 (2.4-2.8) | 1.3 (0.5-2.9) | 0.14 | 6.9 (3.8-10.7) | 6.8 (4.2-11.3) | 0.93 |
| Median household income USD (SD) †* | 94,467 (79,854) | 95,997 (28,981) | 0.81 | 62,542 (38,654) | 56,060 (30,996) | 0.1 |
| Proportion renters, %(IQR)†** | 14.5 (14.5-17.7) | 19.1 (11.2-29.7) | 0.2 | 68.9 (50.4-86.4) | 69.6 (53.6-83.9) | 0.88 |
| Proportion resided in the same house one year ago, % (IQR)†** | 93.9 (93.9-93.9) | 92.9 (90.3-95) | 0.59 | 88.9 (85.4-91.5) | 89.4 (86.8-91.7) | 0.57 |

SD = standard deviation

IQR = Interquartile Range

* characteristics presented as mean (SD) over ZCTAs that belong to specific region

** characteristics presented as median (IQR) over ZCTAs that belong to specific region

† ZCTA characteristic from ACS-2013 (American Community Survey, 5-year estimates: years 2009-2013)

^a^ADI: Area Deprivation Index in 2015 (calculated using ACS 2011-2015 5-year average of 17 socioeconomic disadvantage measures, such as income, education, employment, and housing quality). Lower ADI – less vulnerable population. University of Wisconsin School of Medicine and Public Health. Downloaded from https://www.neighborhoodatlas.medicine.wisc.edu/ 4.10.2024. [Neighborhood Atlas - Download Data (wisc.edu)](https://www.neighborhoodatlas.medicine.wisc.edu/download)

^b^Over-crowded households defined as housing units with more than one occupant per room. Denominator is the number of occupied housing units
