# Supplementary material for: Long-term impacts of hurricanes on mortality among Medicare beneficiaries: evidence from Hurricane Sandy
Source: Front Public Health. 2025 Aug 6;13:1523941. doi: 10.3389/fpubh.2025.1523941 (PMC12364919; doi:10.3389/fpubh.2025.1523941)
Supplement: Supplementary file 2 [file Table_2.docx]

Supplementary Material

**Supplementary Table 2.** Sequential models of adjusted mortality rate ratios

| **Model Type** | **Controlled Covariates** |
| --- | --- |
| 0 | Flood |
| 1 | Flood, Age, Proportion over 65, Proportion Female |
| 2 | Flood, Age, Proportion over 65, Proportion Female, Charlson |
| 3 | Flood, Age, Proportion over 65, Proportion Female, Charlson, Proportion White |
| 4 | Flood, Age, Proportion over 65, Proportion Female, Charlson, Proportion White, Area Deprivation Index, Proportion Overcrowded, Median Household Income |
| 5 | Flood, Age, Proportion over 65, Proportion Female, Charlson, Proportion White, Area Deprivation Index, Proportion Overcrowded, Median Household Income, Proportion Renters |
| 6 | Flood, Age, Proportion over 65, Proportion Female, Charlson, Proportion White, Area Deprivation Index, Proportion Overcrowded, Median Household Income, Proportion Renters, Proportion lived in same house 1 year ago |
| 7 | All time-varying covariates + weights |
| 8 | All baseline covariates + weights |

| **Model Type** | **Adjusted Mortality Rate Ratios** | | |
| --- | --- | --- | --- |
| **All ZCTAs** | **RR** | **95% CI** | **P-value** |
| 0 | 1.18 | 1.15-1.22 | **<0.0001** |
| 1 | 1.17 | 1.13-1.20 | **<0.0001** |
| 2 | 1.13 | 1.10-1.16 | **<0.0001** |
| 3 | 1.13 | 1.10-1.16 | **<0.0001** |
| 4 | 1.11 | 1.07-1.14 | **<0.0001** |
| 5 | 1.09 | 1.05-1.12 | **<0.0001** |
| 6 | 1.09 | 1.06-1.12 | **<0.0001** |
| 7 | 1.09 | 1.06-1.12 | **<0.0001** |
| 8 | 1.09 | 1.06-1.12 | **<0.0001** |
| **New Jersey** |  |  |  |
| 0 | 1.05 | 1.00-1.11 | **0.0303** |
| 1 | 1.13 | 1.08-1.18 | **<.0001** |
| 2 | 1.03 | 0.99-1.08 | 0.1525 |
| 3 | 1.01 | 0.96-1.06 | 0.7547 |
| 4 | 0.97 | 0.93-1.02 | 0.2840 |
| 5 | 0.99 | 0.94-1.04 | 0.5757 |
| 6 | 0.99 | 0.94-1.04 | 0.7203 |
| 7 | 0.99 | 0.94-1.03 | 0.5911 |
| 8 | 1.01 | 0.97-1.06 | 0.6428 |
| **Connecticut** |  |  |  |
| 0 | 1.23 | 1.12-1.35 | **<.0001** |
| 1 | 1.23 | 1.12-1.34 | **<.0001** |
| 2 | 1.21 | 1.10-1.32 | **<.0001** |
| 3 | 1.37 | 1.25-1.50 | **<.0001** |
| 4 | 1.35 | 1.27-1.49 | **<.0001** |
| 5 | 1.28 | 1.15-1.41 | **<.0001** |
| 6 | 1.27 | 1.15-1.41 | **<.0001** |
| 7 | 1.18 | 1.08-1.30 | **0.0004** |
| 8 | 1.19 | 1.09-1.31 | **0.0002** |
| **New York (excluding NYC)** |  |  |  |
| 0 | 1.10 | 0.94-1.28 | 0.2201 |
| 1 | 0.90 | 0.77-1.04 | 0.1529 |
| 2 | 0.90 | 0.78-1.05 | 0.1826 |
| 3 | 0.98 | 0.84-1.14 | 0.7699 |
| 4 | 0.96 | 0.83-1.12 | 0.6195 |
| 5 | 1.01 | 0.86-1.17 | 0.9428 |
| 6 | 1.00 | 0.86-1.17 | 0.9716 |
| 7 | 0.97 | 0.87-1.08 | 0.5340 |
| 8 | 0.96 | 0.86-1.07 | 0.4766 |
| **NYC** |  |  |  |
| 0 | 1.19 | 1.11-1.27 | **<.0001** |
| 1 | 1.18 | 1.11-1.26 | **<.0001** |
| 2 | 1.19 | 1.12-1.28 | **<.0001** |
| 3 | 1.15 | 1.08-1.23 | **<.0001** |
| 4 | 1.19 | 1.11-1.27 | **<.0001** |
| 5 | 1.17 | 1.09-1.25 | **<.0001** |
| 6 | 1.16 | 1.09-1.25 | **<.0001** |
| 7 | 1.10 | 1.04-1.17 | **0.002** |
| 8 | 1.08 | 1.02-1.15 | **0.0089** |
