# Supplementary material for: Long-term impacts of hurricanes on mortality among Medicare beneficiaries: evidence from Hurricane Sandy
Source: Front Public Health. 2025 Aug 6;13:1523941. doi: 10.3389/fpubh.2025.1523941 (PMC12364919; doi:10.3389/fpubh.2025.1523941)
Supplement: Supplementary file 3 [file Data_Sheet_1.docx]

Supplementary Material

**Supplementary Figure 1.** Exclusion cascade depicting the process of creating the cohort of Medicare beneficiaries for this study.

| **Medicare beneficiaries from 20% random sample, reside in NY, NJ, CT in 2012, N=802,968**   \|  \| \| --- \| |  |  |  |  |  |  |  |  |
| --- | --- | --- | --- | --- | --- | --- | --- | --- | --- |
|  |  |  |  |  |  |  |  |  |
|  |  |  |  |  |  |  |  |  |
|  |  |  |  |  |  |  |  |  |
| 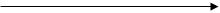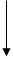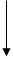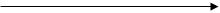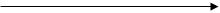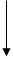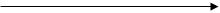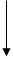  **age in 2012 < 65 (n=131,017)**  **450,037**  **671,951**  **Without Medicare coverage in 2012-2017, change ZCTA during 2013-2017 (n=147,777)**   \|  \| \| --- \|   **Did not reside in specific list of ZCTAs in 2012* (n=221,914)**  **Died in 2012 (n=3,989)**  **302,260**  **298,271** |  |  |  |  |  |  |  |  |
|  |  |  |  |  |  |  |  |  |
|  |  |  |  |  |  |  |  |  |
|  |  |  |  |  |  |  |  |  |
|  |  |  |  |  |  |  |  |  |
|  |  |  |  |  |  |  |  |  |
|  |  |  |  |  |  |  |  |  |
|  |  |  |  |  |  |  |  |  |
|  |  |  |  |  |  |  |  |  |
|  |  |  |  |  |  |  |  |  |
|  |  |  |  |  |  |  |  |  |
|  |  |  |  |  |  |  |  |  |
|  |  |  |  |  |  |  |  |  |
|  |  |  |  |  |  |  |  |  |
|  |  |  |  |  |  |  |  |  |
|  |  |  |  |  |  |  |  |  |
|  |  |  |  |  |  |  |  |  |
|  |  |  |  |  |  |  |  |  |
|  |  |  |  |  |  |  |  |  |
|  |  |  |  |  |  |  |  |  |
